# From the woods to the halls of science: Louis Bernatchez’s contributions to science, wildlife conservation and people

**DOI:** 10.1111/eva.13043

**Published:** 2020-07-14

**Authors:** Anne‐Laure Ferchaud, Martin Laporte, Maren Wellenreuther

**Affiliations:** ^1^ Institut de Biologie Intégrative et des Systèmes (IBIS) Université Laval Québec QC Canada; ^2^ School of Biological Sciences The University of Auckland Auckland New Zealand; ^3^ The New Zealand Institute for Plant and Food Research Ltd Nelson New Zealand

## 1 BIBLIOGRAPHY: AN INTRODUCTION

Louis Bernatchez has been the Editor‐in‐Chief of *Evolutionary Applications* since the beginning of the journal in 2008, and he celebrated his 60th birthday on May 2019. For this occasion, *Evolutionary Applications* has produced a Special Issue to celebrate his accomplishments in applying evolutionary concepts to diverse fields (e.g. wildlife management, medicine, agriculture, aquaculture, forestry, conservation, environmental sciences, microbiology and toxicology) and for his far‐reaching influence on people. The Special Issue features 25 papers that have been authored by 35 former students and postdocs trained in Louis’ research group at Université Laval in Québec, Canada. These alumni together showcase Louis’ wide and diverse impact on raising the next generation of scientists, not merely from a scientific point of view, but also as a mentor, who took great care of the future of his students who were about to take the next step in their career.

**FIGURE 1:**
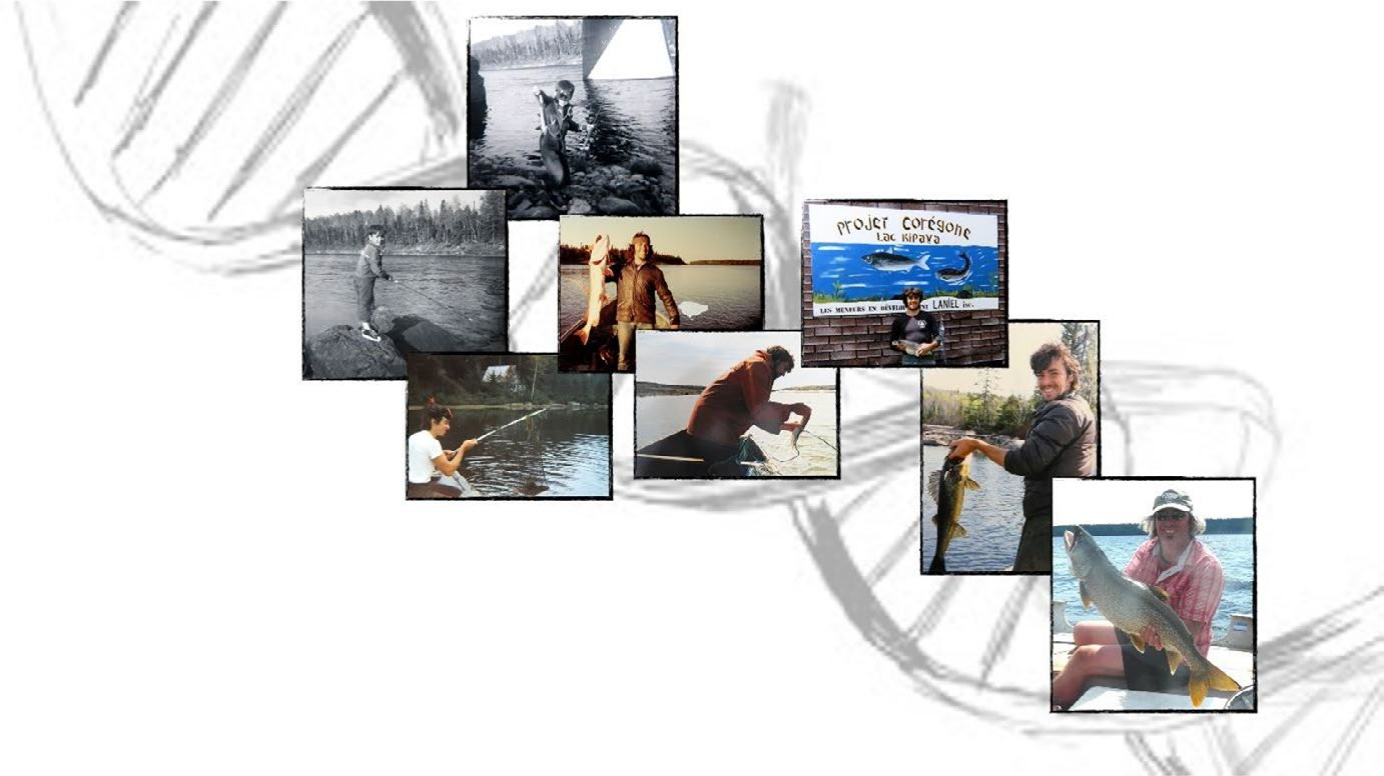
Fishing is an activity that has played a significant role in Louis’ life since an early age. What is clear from these photographs that depict Louis over time, first as a young boy and later when he was a Professor at Laval University, is that it was never the size of the fish that he would catch that mattered most to him, but rather that it was about spending time in nature

Louis was born in 1960 into a family of three children, as the youngest child. He was raised alongside his two older sisters in the small 150‐people community of Lac‐Frontière in Québec, in the St. John River woodland on the border of Maine, USA, where the days were spent outdoors observing nature and going hunting and fishing (Figure 1). At that time, no one could have imagined that this child from the remote countryside would end up in the halls of academia and would pioneer new fields of science. Unlike for some of us, Louis’ path into academia was not inspired by social influences but rather came from a strong and deep connection with nature, something that always stayed with him throughout his career. At the age of 12, Louis remembers, he already knew that he wanted to work as a biologist.

He registered at the Université Laval in Québec and ticked Biology as the only option, not giving any thoughts to secondary choices. He was selected and studied Biology towards a BSc degree. At this time, Louis’ scientific interest in fish was still relatively dormant (other than angling for them!), but this interest grew after his first summer university job working on a research project on migratory movement of Brook Trout (*Salvelinus fontinalis*) in a remote ecological reserve in Québec. His second summer university job brought him to the Cree community of Eastmain, along the eastern coast of James Bay, Québec, where he monitored fish populations that were impacted by a hydropower dam development. Louis spent a total of 11 months among the Cree community of Eastmain. Indeed, after his third undergraduate year at San José State, California, he began an MSc degree, supervised by Prof. Julian Dodson, still working in the Cree community on a project to compare the energetic cost of reproductive migration between anadromous Lake Whitefish (*Coregonus clupeaformis*) and Cisco (*C. artedii*) combining telemetry and respirometry (Bernatchez & Dodson, 1985; Dodson, Lambert, & Bernatchez, 1985). His work on whitefish migration stimulated him to publish a meta‐analysis to investigate the general relationship between bioenergetics and behaviour in anadromous fish migrations (Bernatchez & Dodson, 1987). This first encounter with whitefish turned out to become a lifelong love story for Louis, and he devoted 30 years of his career to the pursuit of understanding adaptive divergence in the sympatric Lake Whitefish species pairs. After his MSc degree, he took an academic break and moved to Laniel, another remote community in western Québec wilderness, to help launch a project to harvest whitefish commercially, which led to the setup of a local start‐up for the production of whitefish caviar! He found himself living among 65 other people, a place even smaller than his childhood community of Lac‐Frontière, something he never imagined would happen. It was at around this time that Louis had made a decision, and that was to become a researcher specializing in fish biology. It is also during that time that he developed his interest for fish photography which led to the publication of the Guide to Freshwater Fishes of Québec and Eastern Canada (Bernatchez & Giroux, 1991).

In 1990, he obtained his PhD degree co‐supervised by Prof. Dodson and Dr Dominick Pallotta, where he was tasked with studying the genetic population structure, once more on coregonine fishes. Louis saw a great deal of creative room in this project and twisted the focus of the project to one that looked into large‐scale phylogeography in these early days of this exciting new discipline of research founded by Prof. John Avise of whom he quickly became a big fan and inspired his work during many years (Avise et al., 1987). After his PhD, he was a postdoc at the Université of Montpellier II in France, in the laboratory of Prof. François Bonhomme, and from 1991 to 1992 a postdoc at the University of Guelph in Canada, collaborating with Profs Moira Ferguson and Roy Danzmann. After these research stays, he returned closer to home again and moved from 1992 to 1995 to the Université du Québec as a Research Associate and subsequently Assistant Professor at the Institut National de la Recherche Scientifique (INRS). Finally, in 1995 he was offered a position at Université of Laval to carry on with his curriculum and became full professor in 2004. Since 2001, he has been the holder of a Canadian Research Chair in Genomics and Conservation of Aquatic Resources. During those years, he has been a visiting research fellow at the University of Brisbane in Australia working with Prof. Craig Moritz (2000–2001), the University of Konstanz in Germany where he was hosted by Prof. Axel Meyer (2002), and then Flinders University in Adelaide (Australia) to work on a long‐term collaborative project with Prof. Luciano Beheregaray and University of British Columbia (Vancouver) with Prof. Eric Taylor in 2017.

During these moves and visits, he managed to stay extremely productive, in both his private and his professional life. He started a family and raised four children who would come along on some of his journeys, for example to Australia, where they shared their time between downtown Brisbane and a beach house lent by Prof. Moritz. He has also contributed immensely to the growth of new disciplines in science. Most notably, Louis has been an early pioneer of phylogeography, and in applying functional genomics to the study of nonmodel organisms. He was also among the very first researchers in the field to stress the importance and benefits of integrating the use of molecular genetics and ecology in studying the processes of adaptive divergence and speciation (Bernatchez, Chouinard, & Lu., 1999). He has also championed the field of population genomics and the integration of genomic, transcriptomic and epigenomic data into a holistic framework alongside ecological, physiological, life‐history and population historical components of adaptation. While fish are his passion, he has not shied away from venturing further, to study other organisms, including many invertebrates, birds and mammals, from oyster, albatross to moose! Overall, Louis’ passion and dedication to understanding biodiversity, most notably, the process of how one species can split into ecotypes and with time into species, and the genetic conservation of aquatic resources, has significantly advanced these fields. Not surprisingly, many scholars in these fields now associate the notions of ecotype divergence, genetic health and evolutionary adaptive potential with his achievements in these areas.

Louis has received numerous honours for his contributions to the scientific advancements of manifold fields. In addition to his Canadian Research Chair, some highlights include the E.W.R. Steacie Award from NSERC (2002), being elected as a member of the Royal Society of Canada (2011) and a Fellow of the American Association for the Advancement of Science (2011), the Prix du Québec, Marie‐Victorin (2012) and invited membership of Faculty Row's Super Professors (2013). In 2016, he was also awarded the Molecular Ecology Prize and selected into the Hall of Excellence, Genetics Section, of the American Fisheries Society (Hansen & Rogers, 2017).

## 2 THE MENTOR, EDITOR AND COLLEAGUE

The concept of a mentor, indeed the word itself, can be traced at least as far back as Homer's *Odyssey*. According to ancient Greek history, the wisdom goddess Athena took the form of a man called Mentor to assume the guardianship of the young prince Telemachus, while his father, Odysseus, was away fighting the Trojan War. Athena's Mentor was not only Telemachus's protector, but also his educator and guide. As Mentor, the goddess encouraged Telemachus to stand up against his mother's suitors and go abroad to find out what had happened to his father. Because of Mentor's relationship with Telemachus, which allowed Athena to provide encouragement and practical advice, the name Mentor was adopted into Latin and other languages, including English. The meaning of a mentor is now synonymous with someone who imparts wisdom to and shares knowledge with a less‐experienced person.

Having a good mentor early in your career can mean the difference between success and failure. Louis has for a long time run a big and active research group, which has required extensive hands‐on training of the graduate students, postdoctoral research fellows and research professionals who have worked with him. He is a dedicated, energetic supervisor with an endless reservoir of energy, who fosters an inspirational laboratory dynamic and where collaboration, within and outside the laboratory, is seen as part of the training. The impressive flow of people who have been through his laboratory has meant that mentoring has been very much part of Louis’ daily life as a scientist, and something in which he has always taken great pride. Louis’ vision is prized for looking beyond short‐term results, showing concern for the members of his laboratory and helping them to progress with their projects, from fixing a recalcitrant experiment, and helping with fieldwork, to organizing international conferences. One of the key characteristics of a good mentor is to show interest and enthusiasm for the various projects and to give due credit for achievements and new ideas. Anyone who has worked with Louis would have experienced the infectious enthusiasm that he can develop for a new research project, something that fuels people to go the extra mile and to achieve beyond the original objectives that were laid out. Louis clearly inspires his students to continue in science, with the majority of his graduates pursuing successful careers either in academia, government or in applied positions in science. In fact, over 150 graduate students and postdocs and about the same number of research assistants and undergrad trainees from all continents have emerged from his laboratory, with many of them working as professors/instructors worldwide, in eight different countries. His laboratory has also been immensely productive, both from a purely academic but also very applied point of view, and he has produced 500 submitted and/or published scientific papers and 145 technical reports at the time of writing, with a continuing trend of strong growth.

Anyone who has interacted with Louis can attest to him being a friendly and accessible person, with a very well‐developed sense of humour. Not only has that energy been used to nurture people in his laboratory, he has also provided extensive services to the scientific community. Many readers will have been in contact with him in his editorial capacities, which have been manifold. He has been working for over 25 years for Wiley in various capacities, such as when he was an Associate Editor of *Evolution* and the *Journal of Evolutionary Biology*. He also served for *Molecular Ecology* as an associate editor from 1995 to 2018 for which he has been the Reviews Editor during 15 years which led to the publication of 150 invited reviews. Since 2008, he has also served as the Editor‐in‐Chief of *Evolutionary Applications* and is a key force behind the success of this journal. It is characteristic of Louis’ editorial work that he maintains a deep respect for the submissions, because he understands that behind many of the manuscripts he handles lies a budding scientist who may be experiencing the peer‐review process for the first time. Most recently, Louis has founded the journal *Environmental DNA*, where he acts also as the Editor‐in‐Chief.

Louis is well known for being a fervent collaborator, and this quality cannot be overstated as being able to work in large teams is nowadays a critical skill. An examination of the 2.4 million scientific articles produced by the top 110 universities in the United States between 1981 and 1999 reveals that research team size in the life sciences increased by around 50% (Vermeulen, Parker, Parker, & Penders, 2013). This is consistent with the view that the last decades of science show a shift from single‐investigator “little science” to increasingly large, expensive, multinational, interdisciplinary and interdependent “big science.” Louis has experienced this shift first‐hand and has succeeded in establishing very large collaborative teams that successfully delivered on science projects. This success in shifting from small to large collaborations can be in large attributed to his desire for interdisciplinary and integrative approaches, and for being generous with good ideas and suggestions. This has been driven by the realization that the quality and the creativity of the science product will be enhanced when joining forces with other scientists, since such unions allow the answering of questions that none could address fully on their own. There is of course no single recipe for making such large collaborative efforts work, but having an eye for designing rigorous science projects and being able to provide constructive feedback on new ideas are crucial skills, and Louis is well known for possessing these skills among his colleagues.

In parallel with the increase in the size of research groups, there has also been an increase in the representation of scientists from different countries in these groups. Indeed, it is not uncommon these days to have different specialists from several countries on a science project, and this has also been the case for many of Louis research collaborations. Louis has strong and ongoing research projects with scientists residing in northern and central Europe, Australia, New Zealand, China, Iran and South America, to name just a few, and has been active on several large project groups, for example The Europeans Union “AquaTrace” project, which seeks to develop tools for tracing and evaluating the genetic impact of fish from aquaculture, and some large Genome Canada projects, such as EPIC‐4, which seeks to support the sustainability of Pacific salmon fisheries and aquaculture industries. In 2019, Louis has also become the main leader of an extensive project sustained by Genome Canada and Genome Québec. The project FISHES (Fostering Indigenous Small‐scale fisheries for Health, Economy, and Food Security) involves numerous university and government scientists as well as partners from over 20 indigenous organizations from three nations (Cree, Inuit and Déné) to develop and apply genomic approaches in concert with Traditional Ecological Knowledge (TEK) to address critical challenges and opportunities related to food security and Commercial, Recreational, and Subsistence (CRS) fisheries belonging to the northern Indigenous peoples in Canada. This most recent large research project draws a circle and brings Louis back to the beginnings where it all started and where he began to develop a deep passion for fish biology along the coast of James–Hudson Bay.

## 3 CONTRIBUTIONS IN THIS SPECIAL ISSUE

**FIGURE 2:**
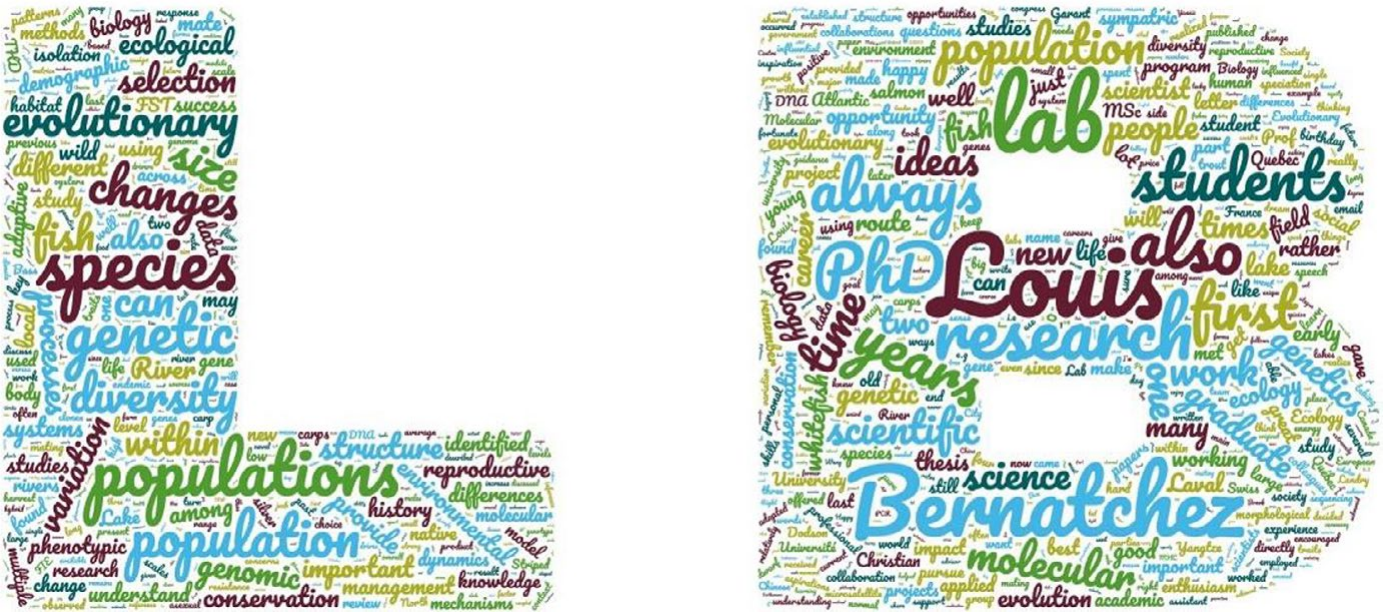
Word clouds compiled from the abstracts (a) and the personal reflection boxes (b) of all authors who contributed to this Special Issue

To illustrate Louis’ impact, we asked several of the alumni that attended his research group over the years (Table 1) to contribute a paper on their present work, including a career reflections box (entitled Box 1 in each contributed paper) to detail how Louis has influenced their career path as a scientist. The authors invited to contribute to this Special Issue include both junior and senior researchers in the broad field of evolutionary/ecological/conservation genomics, reflecting the persistent mentoring to which Louis has constantly devoted so much time and energy (Figure 2). It is worthwhile to note that several of the papers detail long‐term research projects (Bowles, Marin, Mogensen, MacLeod, & Fraser, 2020; Garant, 2020; Perrier, Rougemont, & Charmantier, 2020; Stanford, Clake, Morris, & Rogers, 2020; Veliz et al., 2020), or collective advancements of a research group (Blanchet et al., 2020; Dalziel et al., 2020; Østbye et al., 2020), along with new “perspectives” and approaches in the field (Angers, Perez, Menieucci, & Leung, 2020; Durand et al., 2020; Filteau & Derôme, 2020; Gagnaire, 2020; Hallin et al., 2020; Lu et al., 2020; Milot, Béchet, & Maris, 2020; Sutherland et al., 2020), and the use of genomics information for wildlife management and conservation (Bangs, Douglas, Brunner, & Douglas, 2020; Bourret, Albert, April, Côté, & Morissette, 2020; Capblancq, Després, & Mavárez, 2020; Delrieu‐Trottin et al., 2020; Leblanc et al., 2020; Uusi‐Heikkilä, 2020; Young, Cluney, & Weir, 2020) as well as human health (Wirth, Wong, Vandenesch, & Rasigade, 2020).

**TABLE 1:** List of alumni (Names) and current members of Louis' laboratory who contributed to the Special Issue, alongside members of their own current research group

Names	Status	Year	Current position
Vicky Albert	M.Sc.	2005	Biologist, Ministère des Forêts, de la Faune et des Parcs, Québec, QC, Canada
Bernard Angers	Ph.D.	1997	Professor, Département des Sciences biologiques, Université de Montréal, QC, Canada
Julien April	Ph.D.	2012	Biologist, Ministère des Forêts, de la Faune et des Parcs, Québec, QC, Canada
Simon Blanchet	Ph.D.	2007	Researcher, Station d'Écologie Théorique et Expérimentale du CNRS à Moulis, Université Toulouse III Paul Sabatier, France
Vincent Bourret	Ph.D.	2013	Biologist, Ministère des Forêts, de la Faune et des Parcs, Québec, QC, Canada
Patrick Brunner	Ph.D.	1997	Professor, ETH Zurich ‐ Integrative Biology Universitätstrasse, Zurich, Switzerland
Vincent Castric	Ph.D.	2002	Researcher, CNRS, Univ. Lille, UMR 8198 ‐ Evo‐Eco‐Paleo, Lille, France
Guillaume Côté	M.Sc.	2007	Biologist, Ministère des Forêts, de la Faune et des Parcs, Québec, QC, Canada
Anne Dalziel	Postdoc	2015	Assistant Professor, Department of Biology, Saint Mary's University, NS, Canada
Nicolas Derôme	Postdoc	2007	Associate Professor, Département de biologie, Université Laval, QC, Canada
Anne‐Marie Dion‐Côté	Ph.D.	2016	Assistant Professor, Département de biologie, Université de Moncton, NB, Canada
Marlis Douglas	Ph.D.	1997	Endowed Professor, Biological Sciences, University of Arkansas, USA
Anne‐Laure Ferchaud	Postdoc	Ongoing	Postdoctorate, Département de biologie, Université Laval, QC, Canada
Marie Filteau	Postdoc	2012	Adjunt Professor, Département des sciences des aliments, Uniuversité Laval, QC, Canada
Dylan Fraser	Ph.D.	2005	Associate Professor, Department of Biology, Concordia University, QC, Canada
Pierre‐Alexandre Gagnaire	Postdoc	2011	Researcher, ISEM, Université de Montpellier, France
Dany Garant	Ph.D.	2002	Professor, Département de biologie, Université de Sherbrooke, QC, Canada
Nicolas Hubert	Postdoc	2006	Researcher, UMR 5554 ISEM, Université de Montpellier, France
Christian Landry	M.Sc.	2001	Professor, Département de biologie, Université Laval, QC, Canada
Martin Laporte	Postdoc	Ongoing	Postdoctorate, Département de biologie, Université Laval, QC, Canada
Guoqing Lu	Ph.D.	2000	Professor Department of Biology, University of Nebraska at Omaha, Omaha, NE, USA
Jesús Mavárez	Postdoc	2008	Researcher, Laboratoire d’Écologie Alpine, Université Grenoble, France & Associate Professor, Departamento de Ciencias Biológicasy Ambientales, Universidad Jorge Tadeo Lozano, Bogotá, Colombia
Emmanuel Milot	Ph.D.	2009	Professor, Department of Chemistry, Biochemistry and Physics, Université du Québec à Trois‐Rivières, QQC, Canada
Olivier Morissette	Ph.D.	2019	Biologist, Ministère des Forêts, de la Faune et des Parcs, Québec, QC, Canada
Kjartan Østbye	Postdoc	2009	Professor, Department of Biosciences, Centre for Ecological and Evolutionary Synthesis (CEES), University of Oslo, Norway
Scott Pavey	Postdoc	2015	Associate Professor, Department of Biological Sciences, University of New Brunswick, NB, Canada
Charles Perrier	Postdoc	2014	Postdoctorate., CEFE UMR 5175, CNRS, Universit´e de Montpellier, France
Sean Rogers	Ph.D.	2005	Associate Professor Department of Biological Sciences, University of Calgary, AB, Canada
Quentin Rougemont	Postdoc	Ongoing	Postdoctorate, Département de biologie, Université Laval, QC, Canada
Ben Sutherland	Postdoc	2017	Researcher, Pacific Biological Station, Fisheries and Oceans Canada, BC, Canada
Silva Uusi‐Heikkilä	B.Sc.	2006	Senior researcher, Department of Biological and Environmental Science, University of Jyväskylä, Finland
David Véliz	Ph.D.	2005	Professor, Departamento de Ciencias Ecológicas, Universidad de Chile, Santiago, Chile
Laura Weir	Postdoc	2012	Assistant Professor, Department of Biology, Saint Mary's University, NS, Canada
Andrew Whiteley	Postdoc	2009	Associate Professor, Department of Ecosystem and Conservation Sciences and Wildlife Biology, University of Montana, MT, USA
Thierry Wirth	Postdoc	2000	Researcher, Institut de Systématique, Evolution, Biodiversité, Muséum National d’Histoire Naturelle, Paris, France
Glen Yannic	Postdoc	2014	Associate Professor, Univ. Grenoble Alpes, Univ. Savoie Mont Blanc, Grenoble, France + D5:D40

Note

The Table shows the degree they obtained during their time at Louis research group (Status), the year they left the research group (Year) and their current position (Current Position). For a complete list of trainees, see: http://www2.bio.ulaval.ca/louisbernatchez/people.htm

The diversity of topics in this Special Issue showcases Louis’ accomplishments and interests in studies that contribute and develop new evolutionary approaches that can be used to improve and guide the management and conservation of biodiversity in nature. It is this specific focus of science which he passed on to his students and ranges from an understanding of evolutionary processes such as connectivity and species boundaries to wildlife management and aquaculture (Figure 2). Most of the studies presented are based on wild populations, with a great majority of them focusing on fish (Angers et al., 2020; Bangs et al., 2020; Blanchet et al., 2020; Bowles et al., 2020; Dalziel et al., 2020; Delrieu‐Trottin et al., 2020; Gagnaire, 2020; Leblanc et al., 2020; Lu et al., 2020; Østbye et al., 2020; Stanford et al., 2020; Uusi‐Heikkilä, 2020; Veliz et al., 2020; Young et al., 2020), but also other aquatic organisms (Sutherland et al., 2020), as well as birds and mammals (Garant, 2020; Yannic, Hagen, Leugger, Karger, & Pellissier, 2020), terrestrial invertebrates (Capblancq et al., 2020), plants (Durand et al., 2020), yeast (Hallin et al., 2020) and bacteria (Filteau & Derôme, 2020; Wirth et al., 2020).

It is of note that, among these primary research contributions, several of the articles adopt integrative approaches, combining genomic and phenotypic data (Blanchet et al., 2020), and even weaving that in with additional data sets, such as habitat requirements (Capblancq et al., 2020) and indigenous knowledge (Bowles et al., 2020). Østbye et al. (2020), for example, document eco‐morphological and life‐history traits of Arctic Charr (*Salvelinus alpinus*) morphs in addition to a phylogenetic (mtDNA + microsatellites) analysis, and Capblancq et al. (2020) combine genetic, morphological and ecological variables to document factors explaining the level of divergence between a butterfly species pair of *Coenonympha macromma* and *C. gardetta* in a hybrid zone. In the same way, an integrative approach proposed by Yannic et al. (2020) harnesses current and past species distribution modelling, landscape genetic simulations, empirical genetic and fossil data to identify the drivers that shape the current intraspecific genetic diversity in reindeer (*Rangifer tarandus)*. Hallin et al. (2020) demonstrate that while the disciplines of biology are organized at several levels, ranging from molecular biology to ecology, analogy to other biological processes at different organizational levels, for example organismal versus ecological levels, can be found and is inherent. They conclude that therefore the studies seeking to investigate processes operating in different systems could enrich work through a multidisciplinary approach, and by drawing in similarities from other systems. Gagnaire (2020) studies ecological and evolutionary connectivity in varied taxonomic groups at macro‐evolutionary scales, while Blanchet et al. (2020) investigate connectivity patterns of dozens of freshwater fish species, including cyprinid fish assemblages, within dendritic systems using genetic variation inference and ecosystem service simulations to reveal that intraspecific diversity affects community dynamics but also key ecosystems functions such as litter degradation. Angers et al. (2020) go beyond mere DNA variation and discuss the relevance of assessing different sources of epigenetic variation in various exemplar vertebrate species, like the fish species *Chrosomus eos‐neogaeus* and the salamander *Ambystoma laterale‐jeffer,* to better understand phenotypic plasticity and bet‐hedging strategy, something that has received recent broad attention (Leung, Breton, & Angers, 2016; Vogt, 2017).

The understanding of reproductive isolation is another key topic that is being addressed by several authors, for instance, by conducting hybridization studies at the genus level (Delrieu‐Trottin et al., 2020), or by investigating introgression within genera (Bangs et al., 2020; Capblancq et al., 2020). Bangs et al. (2020) study admixture across ten species of Catostomidae across the Colorado River, where habitat alterations are not only accelerating the breakdown of reproductive barriers, but are also promoting the process of introgression. Hybridization occurred at the genus level despite phylogenetic distance, whereas introgression was only detected within subgenera, implicating phylogenetic distance and/or ecological specialization contribute as important drivers of reproductive isolation, a pattern that had been documented previously (Fine, 2015; Sánchez‐Guillén, Córdoba‐Aguilar, Córdoba‐Aguilar, Cordero‐Rivera, & Wellenreuther, 2013). Delrieu‐Trottin et al. (2020) apply DNA barcoding to investigate species delimitation patterns within the Indo‐Australian Archipelago grey mullet taxonomic complex, a notorious case of taxonomic complexity that requires DNA‐based identification methods given that traditional morphological identifications are usually not repeatable and sequence mislabelling is frequent in international sequence repositories. Lu et al. (2020) review the evolutionary history of silver and bigheads carps (*Hypophthalmichthys molitrix* and *H. nobilis*) and discuss the reasons why they rarely hybridize in their native range in China, but show extensive hybridization when invading aquatic systems in North America. Moreover, Dalziel et al. (2020) discuss the usefulness of studying wild, asexual, vertebrate hybrids as they have many characteristics that make them good model systems for studying how genomes evolve and epigenetic modifications influence animal physiology. They find persistent environmental and/or genetic factors that are causing a bias in cross direction, and end up by discussion more broadly the processes that are enabling the transition to asexuality and the potential physiological consequences of epigenetic variation. Durand et al. (2020) review how the recent advancement in self‐incompatibility in *Brassicaceae* could prove novel insights into the emergence, maintenance and diversification of complex genetic systems.

Several of the contributions are highlighting the significant added insights that genomic data can provide, and how this can be applied to inform the conservation and management of aquatic species. Of particular interest is the comparative genomics framework to provide a deeper understanding of evolutionary process connectivity proposed by Gagnaire (2020). Gagnaire's proposed framework relies on coupling the inference of long‐term demographic and selective history with an assessment of the contemporary consequences of genetic connectivity. He then discusses how standardizing this approach across several species occupying the same landscape can help to understand how spatial environmental heterogeneity has shaped the diversity of historical and contemporary connectivity patterns in different taxa with contrasted life‐history traits. Leblanc et al. (2020) used a genotype‐by‐sequencing (GBS) approach to investigate genetic structure of striped bass (*Morone saxatilis*) along the Canadian and USA Atlantic Coast. This study demonstrates the power of GBS to resolve fine‐scale genetic structure, to provide an efficient means to assign fish to their population of origin and document unexpected occurrence of admixture, all of which has important implications for the local and international management of this species.

Other contributions include microbiome research in the context of aquaculture production (Filteau and Derôme 2020), as well as work on fish supplementation (Blanchet et al. 2020; Bowles et al. 2020), forensics and fishery management (Bourret et al. 2020), and the process of domestication in aquaculture (Sutherland et al. 2020), the historical and contemporary admixture process across species (Bangs et al. 2020; Lu et al. 2020), invasive species control (Bourret et al. 2020; Lu et al. 2020), local and international management (Leblanc et al. 2020) and public policies (Bourret et al. 2020; Veliz et al., 2020). Sutherland et al. (2020) analyse a worldwide data set of naturalized and wild populations of Pacific Oyster (*Crassostera gigas*) to understand genetic variation among populations and farmed types and propose appropriate industrial decisions around this major industry in aquaculture. They conclude that implications of potential introgression from hatchery‐farmed oysters depend on whether naturalized populations are valued as a locally adapted resource or as an introduced, invasive species. Bourret et al. (2020) argue that evolutionary biology, through research work linked to conservation, management and forensics, can have a significant impact on wildlife agencies and governmental practices, and provide examples to support this. Specifically, these authors, currently employees for the government's Wildlife Department in Québec, demonstrate how they have been proactive in reducing the “research‐implementation” gap owing to an outstanding collaboration with Louis, which includes work on the management of exploited wildlife species to the evolutionary application in wildlife conservation and forensics. Milot et al. (2020) propose that advocates for the conservation of evolutionary potential should position their conception along four dimensions to determine why and when the maintenance of evolutionary potential is an appropriate target for the conservation of biodiversity. Filteau and Derôme (2020) examined current knowledge concerning factors governing assembly and dynamics of fish hosts and their microbiota, and also discuss the current microbial community manipulation strategies from an evolutionary standpoint to provide a perspective on the potential for risks, conflict and opportunities in aquaculture.

Two contributions focus on human‐induced evolution. First, Garant (2020) reviews research that integrates ecological and evolutionary theories with molecular ecology, quantitative genetics and long‐term monitoring of individually marked wild animals, with a focus on two model species; the tree swallow (*Tachycineta bicolor*) and eastern chipmunk (*Tamias striatus*). He then outlines ongoing research by his group to understand the limits of adaptive potential by determining the factors constraining the evolvability of plasticity. Second, Perrier et al. (2020) investigate genomic processes underlying local adaptation in blue tit (*Cyanistes caeruleus*) inhabiting different habitats, such as neighbouring deciduous and evergreen environments. They specifically identify footprints of local selection but also quantify spatio‐temporal variation of populations’ demography and variation in recombination rate and diversity along the genome. Briefly, they find weak and nonparallel footprints of divergent selection in deciduous and evergreen populations that were consistent with their demography and the probable polygenic nature of local adaptations in these habitats.

Another type of human‐induced evolution can be studied in the form of fisheries‐induced evolutionary change in aquatic animals. Specifically, systems where fisheries differentially exploit phenotypically discrete, age‐invariant life histories provide particularly excellent candidates for detecting fisheries‐induced evolution, and two contributions make that their focus. Young et al. (2020) argue that fishery imposed selection against hook nose in males, a life‐history trait in coho Salmon (*Oncorhynchus kisutch*), drives an evolutionary increase in the proportion of males adopting the jack tactic. They conclude that harvest‐induced genetic changes may arise within 1–2.5 generations in these long‐lived wild fishes, demonstrating the need to investigate concerns about harvest‐induced evolution quickly once they have been raised. Bowles et al. (2020) highlight signatures of fisheries‐induced selection between historical and contemporary samples of wild walleye populations (*Sander vitreus*), which are paralleled both by phenotypic and genomic changes in the three harvested study populations and in both sexes, while no changes could be detected in the reference population. Moreover, Uusi‐Heikkilä (2020) outlines implications of size‐selective fisheries on sexual selection in various species studied by her and other research groups. Rapid environmental changes caused by human activities also impact the global distribution and abundance of species, highlighting the urgency to understand and predict how populations will respond. Here, the analysis of differentially expressed genes has elucidated areas of the genome involved in adaptive divergence to past and present environmental change. Veliz et al. (2020) documented patterns of gene expression and genetic diversity to assess the impact of the implementation of new public policies towards mitigating the detrimental effect of wastewater production on fish health and demography in Chile. Finally, re‐analysing published data set using “pathway analyses,” Stanford et al. (2020) demonstrate the usefulness of such methods to circumvent problems encountered in transcriptomics and posed by the huge numbers of differentially expressed genes and limited knowledge of how these genes work in conjunction with each other.

One last contribution deals with the relevance of genomics to address human health‐related issues, particularly in the context of epidemia where understanding the driving forces underlying such threats to human health is key to inform intervention strategies against it. Here, Wirth et al. (2020) illustrate how the use of the recently described time‐scaled haplotypic density (THD) method can be used to correlate measures of the epidemic success of a pathogen with ancillary parameters such as its drug resistance profile as a flexible tool to identify such driving forces in exemplary epidemics of multidrug‐resistant (MDR) bacterial lineages, such as *Mycobacterium tuberculosis* Beijing, Salmonella Typhi H58 and *Staphylococcus aureus* ST8. Their findings illustrate how THD can help leverage the massive genomic data sets generated by molecular epidemiology studies to address new questions pertaining to human health.

These research contributions together showcase that the future of the field of applied and fundamental work in ecological genomics and biodiversity conservation is in good hands. Louis’ endless input into the training of the next generation of academic offspring has been immensely successful and they are now walking the halls of science with strength and rigour. Not only that, this next generation has also been experiencing first‐hand what good mentorship and teamwork looks like, and they are now in the prime position to extend this network further by extending what they have learned to the future generation of scientists that are passing through their research groups.

Box 1 **Personal reflections of Anne‐Laure Ferchaud**

2014: I was really scared to come into Québec city – new continent, new country, new social network, new laboratory … you know all the uncertainty coming along with postdoc life … especially because I was leaving the happiest country of the world and was not sure what I will find in QC, and on top of that I was (and I am still) really impressed by Louis’ career. Michael (Møller Hansen, my postdoc supervisor at that time) reassured me and said “you will be great in Louis’ laboratory.” Michael: you were truly right!!! Almost 6 years later, I am still here, with no desire to leave! If Louis did not exist, it would be very hard to imagine that such a person could be real! No need here to detail his outstanding research skills (his career, his collaborative network and this Special Issue speak loudly about these). Louis is also a devoted teacher, mentor, laboratory leader, chief editor, institute director … I have learned so much from all his facets actually, but the laboratory leader is certainly the one that I can describe more: Louis is always enthusiastic to start a new project, ahead with a new approach, always asking to go further. One of the many strengths of Louis is certainly to see the bright side of everything and everyone. Louis cares about sciences for sure, but most importantly he cares about people! He is constantly making sure that everyone in his laboratory has everything on hand to make his/her project work, encouraging people to attend international congresses, to prepare for their futures. Organizing a party is almost as important as organizing a laboratory meeting for him, so he can make also sure of everyone's well‐being! Being in Louis’ laboratory is like being in a big scientific family with no borders: once you come in, you never leave the family: you can leave the laboratory but not the family! I have learned so much from all these family members, and I have learned so much from Louis. Louis, you definitely help me to be more confident, ambitious, creative and stronger in sciences, but also in daily life. I feel so fortunate to be in this big family!! Thank you so much Louis for your amazing mentoring, great support and everything else!! BONNE FÊTE LOUIS!!

Photograph for Box 1: Anne‐Laure Ferchaud—Anne‐Laure is a postdoctorate in Louis’ laboratory—Département de Biologie, Univesité Laval, Québec, Canada.

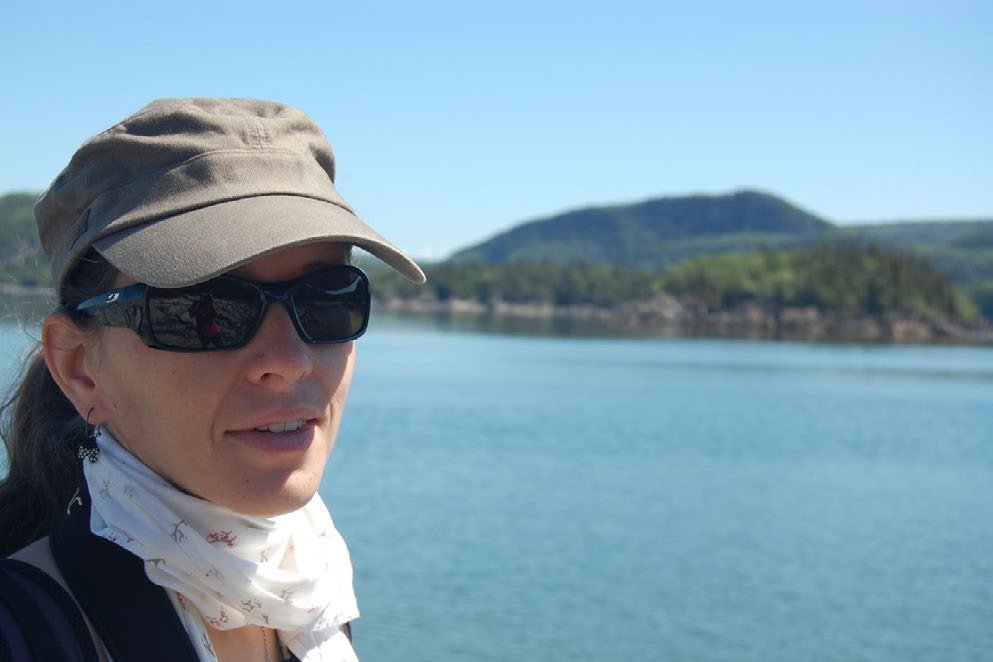

Box 2 **Personal reflections of Martin Laporte**

Back to 2009, when I first met Louis at the Canadian Society of Ecology and Evolution in Halifax. I was a Masters student presenting his results on a new parallel evolution system. Leading the study of the parallel evolution of the lake whitefish, Louis was famous to me. Thus, I spotted him in the room just before my presentation, which I do not remember at all. He talked to me immediately afterwards, and showed genuine interest in my work, but more importantly, in me. Later during the year, he evaluated my Masters dissertation and offered me a PhD project in his laboratory, which I … declined. This was because I had just given my word for another PhD project in Montpellier, France. Well, I don't remember if it was a case of “I kept my word” or if it was the field work in Corsica that drove my decision, but in any case, I said to Louis that he would be my first postdoc position. In 2013, I finally started work in his laboratory and had the chance to lead and collaborate on tremendous projects. To this day, it is a total of 28 scientific papers that Louis has given me the chance to work upon, which encompass many subjects such as quantitative genetics (Laporte et al., 2015), population genomics (Ferchaud, Laporte, Perrier, & Bernatchez, 2018; Laporte, Dalziel, Martin, & Bernatchez, 2016; Laporte, Pavey, et al., 2016), ecophysiology and morphology (Dalziel, Martin, Laporte, Guderley, & Bernatchez, 2015; Laporte, Dalziel, et al., 2016; Laporte, Pavey, et al., 2016), epigenomics (Laporte et al. 2019; Le Luyer et al. 2017), microbiota (Sevellec, Laporte, Bernatchez, Derome, & Bernatchez, 2019) and environmental DNA (Laporte et al. 2020). I could not have dreamt of a better place to learn so many subjects. If I have to describe Louis, I will say that I’m really amazed by his capacity to lead several projects at the same time, his guts to start big and bold projects, his capacity to feel the coming wave of a new trends in the field, and finally how he still cares about people despite the high amount of stress that he has to deal with. Thank Louis for your trust, your understanding, your amazing parties, and also, for always letting your students catch more and bigger fish! On a final note, a big thank you for supporting the hell of a wreck that I was in 2018 …

Bonne fête Louis!

Photograph for Box 2: Martin Laporte—Martin is a postdoctorate in Louis’ laboratory—Département de Biologie, Univesité Laval, Québec, Canada.

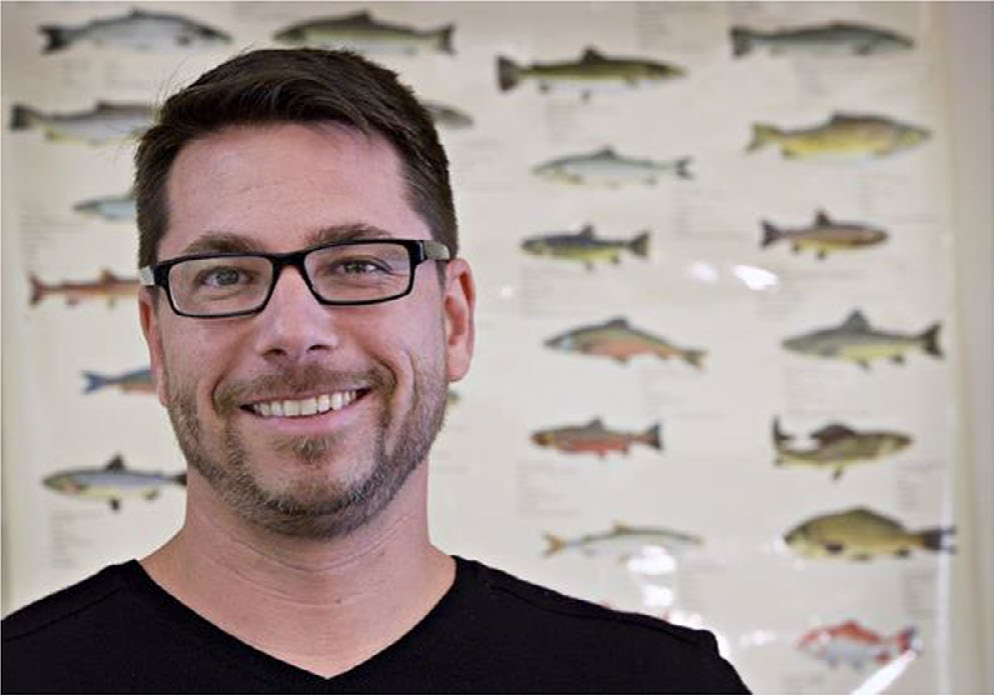

Box 3 **Personal reflections of Maren Wellenreuther**

A life as a scientist is not a straight road. There are various crossroads and steep hills that need to be conquered. The reward for managing to get through these obstacles is captured by moments where one has reached a milestone or a goal, and then you can take a break, sit down, and even if it's just for a short moment, enjoy the glorious views around you. Mentors and sponsors can lend a hand when the hills become steep, yet most of the time, this happens in the background, out of the sight of others, and with little direct reward for the mentor and sponsor. Louis has been the most formative mentor as well as sponsor in my career as a scientist, and I feel truly grateful for the growth opportunities that he has provided me with.

I met Louis for the first time at the conference of the European Society for Evolutionary Biology (ESEB) in Tübingen (Germany) in 2011, and then again at the ESEB conference in Lisbon (Portugal) in 2013. During that time, I was working as a postdoc at Lund University in Sweden, trying to build my own research career as well as care for my young family. The demands on me at that time were immense and I felt that the future of my career in science was hanging on silk threads. For my PhD thesis, I had been studying the adaptive radiation of triplefin fishes (Tripterygiidae) in New Zealand, and so I was well aware of Louis’ foundational work on the adaptive divergence in Lake Whitefish (*Coregonus clupeaformis*) and other species. In my naïve view, however, I imaged that Louis was sitting in a tall ivory tower, out of reach for a young scientist, and so I was ill prepared when I first met him at the conference: he was humble and surprisingly approachable and displayed a keen interest in discussing science without being authoritative about it. I soon realised that Louis and I both shared a very diverse interest in science, from evolutionary genomics to natural history, and that we both felt that applied approaches were fundamental to safeguard the future of this planet. Not surprisingly, we quickly became engaged in energetic and varied discussions and soon after, we started to plan our first projects together.

I can now look back at an uncountable number of projects that I was fortunate to share with Louis, but I will briefly mention the most memorable ones here. I have to start with our 2016 Special Issue in *Evolutionary Applications* on “Women's contribution to basic and applied evolutionary biology” that we co‐edited and compiled (the introductory paper to this Special Issue: Wellenreuther and Otto 2016; featuring personal reflections of Rosemary Grant, Mary Jane West‐Eberhard and Josephine Pemberton). The idea for this Special Issues came from Louis. He was well aware that I had a keen interest in the role of women in science, and that I was the coordinator of the women's STEM network at Lund University (Wellenreuther, Stadmark, Stadmark, & Broström, 2016), and we had countless discussions where argued that while the face of science had changed over time, that women remained still less visible than their male counterparts. Once the idea of this Special Issue was hatched Louis proposed it to Wiley and they were immediately supportive. The overarching goal of the Special Issue was to celebrate the outstanding achievements and contributions of women scientists, and I will be forever grateful that we devoted our time to this and produced a volume of which I will be forever proud.

Another career highlight for me was our work to become joint advocates for a better integration of genomic technologies into the management and sustainable use of aquatic species. Louis has for long been a role model and pioneer for integrating genomics into conservation biology, and I became motivated by him to follow suit. In 2016, we organised a OECD‐funded symposium at the Word Fisheries Congress in South Korea, and then compiled, with our invited speakers, an extremely diverse and detailed summary and synthesis paper (the supplementary material for this paper alone is 172 pages) (Bernatchez et al. 2017) entitled “Harnessing the power of genomics to secure the future of seafood.” My passion for this topic has since become deeply embedded in my research career, and is something that I think will stay with me.

I was the driver for the last project that I will highlight here, which was fuelled by my deep interest in structural genomic variation. For long I had been questioning our approach to describe genomic variation of species, which was predominately only captured by variation in Single Nucleotide Polymorphisms (SNPs). I discussed my thoughts with Louis and convinced him that structural genomic variation has been underappreciated, yet that this type of variation contributes to large amounts (and often even more so than SNP variants) of genomic variation (Catanach, Deng, Deng, Charles, Bernatchez, & Wellenreuther, 2019), and that we have an urgent need to go beyond SNPs in our research. The first outcome of our intense discussions on this topic was a review paper on eco‐evolutionary genomics of chromosomal inversions (Wellenreuther and Bernatchez 2018). We then started several projects on the topic of structural variants, from studies on the adaptive role of inversion polymorphisms, symposia at conferences and a Special Issue in *Molecular Ecology* entitled “The role of structural genomic variants in adaptive evolution and species diversification” (Wellenreuther, Mérot, Mérot, Berdan, & Bernatchez, 2019). This interest is still going strong and has become strong research interest in both of our research groups (Mérot et al. 2018; Mérot et al., 2019).

But what has been more important than working on these science projects with Louis, has been his tireless role as my sounding board, and being my mentor and sponsor. He has helped me to navigate the crossroads of a career in science. One of the most important changes in my way of thinking that I credit to him relates to my view of “applied science.” Coming from a strict academic background, these words were often uttered at the universities with some sort of disrespect, and all too commonly associated with a label of second‐class science. Louis taught me that the Holy Grail in science is to apply the best scientific tests to topics that matter in this world, and that the translation of scientific findings into applications is something that truly matters, and for this I will be forever grateful. Thank you Louis for all your support and persistence, for your never ending energy to argue with me about small and big things, and for teaching me always to put people first.

Photograph for Box 3: Maren Wellenreuther. Maren is a Science Group The New Zealand Institute for Plant & Food Research in Nelson (New Zealand) and an associate professor at the University of Auckland (New Zealand).

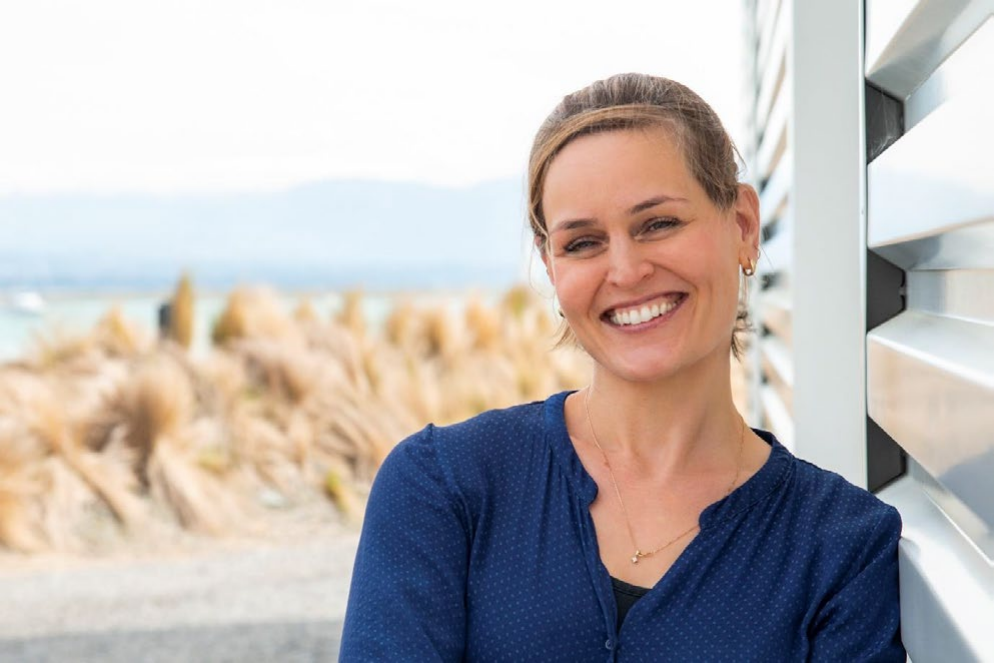

## CONFLICT OF INTEREST

None declared.
